# Supplementary material for: Mechanisms governing the pioneering and redistribution capabilities of the non-classical pioneer PU.1
Source: Nat Commun. 2020 Jan 21;11:402. doi: 10.1038/s41467-019-13960-2 (PMC6972792; doi:10.1038/s41467-019-13960-2)
Supplement: Supplementary file 7 — Source data [file 41467_2019_13960_MOESM7_ESM.zip › Source_Data/Figure6/Figure6B_MotifScanOutput/FLI1_mutPU.1/homerResults/motif8.similar.html]

motif8

## Information for motif8

T
C
A
G
G
C
A
T
C
T
A
G
A
C
T
G
C
T
A
G
A
C
T
G
A
C
T
G
G
T
C
A
C
T
A
G
C
A
T
G
  
Reverse Opposite:  

G
T
A
C
G
A
T
C
C
A
G
T
G
T
A
C
A
G
T
C
G
A
T
C
G
T
A
C
A
G
T
C
C
G
T
A
A
G
T
C
  

|  |  |
| --- | --- |
| p-value: | 1e-135 |
| log p-value: | -3.130e+02 |
| Information Content per bp: | 1.703 |
| Number of Target Sequences with motif | 2542.0 |
| Percentage of Target Sequences with motif | 24.78% |
| Number of Background Sequences with motif | 5382.7 |
| Percentage of Background Sequences with motif | 14.12% |
| Average Position of motif in Targets | 100.9 +/- 54.4bp |
| Average Position of motif in Background | 100.5 +/- 61.4bp |
| Strand Bias (log2 ratio + to - strand density) | -0.1 |
| Multiplicity (# of sites on avg that occur together) | 1.19 |
| Motif File: | file (matrix) reverse opposite |

### Similar de novo motifs found

|  |  |  |  |  |  |  |  |
| --- | --- | --- | --- | --- | --- | --- | --- |
| Rank | Match Score | Redundant Motif | P-value | log P-value | % of Targets | % of Background | Motif file |
| 1 | 0.968 | G A T C G A T C C A G T A G T C G T A C G A T C G T A C A G T C C G T A A T G C G T C A A G T C | 1e-112 | -259.999347 | 28.94% | 18.43% | motif file (matrix) |
| 2 | 0.795 | C A T G C A T G T C A G T C A G T C A G T C A G C T A G T C A G | 1e-95 | -219.985237 | 46.28% | 34.98% | motif file (matrix) |
| 3 | 0.785 | A C T G A C T G T C A G T A C G T C A G C T A G A C T G | 1e-90 | -208.207415 | 47.06% | 36.03% | motif file (matrix) |
| 4 | 0.943 | A C T G T A C G G C A T A C T G A C T G A C T G A C T G A C T G C G T A A C T G A C T G C T G A C A T G | 1e-60 | -139.228421 | 9.54% | 4.97% | motif file (matrix) |
| 5 | 0.948 | C T A G C A G T A T C G G C A T C T A G C A T G C T A G C A T G C T A G G C T A C T A G C A T G T A C G T C A G | 1e-55 | -128.124506 | 8.70% | 4.51% | motif file (matrix) |
